# Promoting Awareness of the Role of the District Clinical Specialist Team in the Amathole District, South Africa: A Valuable Specialty in Improving Healthcare Access and Quality

**DOI:** 10.3390/tropicalmed7120436

**Published:** 2022-12-13

**Authors:** Oyebola G. Oyebanji, Thubelihle Mathole, Debra Jackson

**Affiliations:** School of Public Health, University of the Western Cape, Cape Town 7535, South Africa

**Keywords:** PMTCT, HIV, South Africa, District Clinical Specialist Team, quality improvement

## Abstract

Introduction: This study explored the understanding of healthcare professionals on the role of the District Clinical Specialist Team (DCST) and how the team works together with the district personnel at different management levels to improve and strengthen the Prevention of Mother-to-Child Transmission of HIV (PMTCT) programme performance across four sub-districts in the Amathole district of the Eastern Cape Province, South Africa. Methods: An interpretive qualitative case study was used to understand the role played by the DCST in improving PMTCT programme performance in the district. We used a purposive sampling method to select eight participants involved in providing technical assistance to support the implementation of the quality improvement programme. We conducted in-depth interviews with all the participants; all were females in their mid-forties. Data were analysed thematically by identifying themes and reporting patterns within the data. Findings: Most interviewees were females in their mid-forties and had been at their respective facilities for at least five years. The findings were discussed based on three themes: capacity building, programme performance oversight and monitoring, and technical support. The DCST significantly enhances the staff’s clinical skills, knowledge, and work performance to care for and manage the mother and baby pair. In addition, the DCST plays a vital role in providing programme oversight and complements the technical support provided by the Department of Health (DoH) managers and the quality improvement programme support by the South to South (S2S) team aimed at improving and achieving the PMTCT programme’s desired outcomes. The DCST also provided additional support for data verification to identify gaps in the PMTCT programme. Conclusion: The role of DCST is essential in improving the quality and service provision of the PMTCT programme and is critical to assist the team at different levels in addressing challenges encountered and training and mentoring the needs of the staff. In addition, DCST’s responsibilities cannot be fully achieved without a good working relationship with the quality improvement and district health teams because they work better together to ensure that the programme is performing optimally. Take-home message: This study showed that the District Clinical Specialist Team is vital for improving the quality and service provision of the PMTCT programme and it is essential for addressing challenges encountered by healthcare facilities and the staff providing PMTCT services.

## 1. Introduction

In 2012, South Africa established district-based clinical specialist teams (DCSTs), a re-engineering stream in Primary Health Care (PHC) created to facilitate clinical governance at the lowest levels of the healthcare system. The DCST role involved ensuring that health facilities across all levels are responsible for the continuous improvement of quality care and service delivery for effective and efficient delivery of universal healthcare to individuals and communities [1,2,3]. One of South Africa’s National Department of Health (DoH) partners, the South to South Program for Comprehensive Family HIV Care and Treatment, Stellenbosch University, Cape Town, South Africa (S2S), worked in the Amathole district and complemented the technical assistance provided by the DCST. As a result, S2S implemented a quality improvement collaborative model to eliminate Mother-to-Child Human Immunodeficiency Virus (HIV) transmission (eMTCT) in one hundred and twelve facilities across four sub-districts (Amahlathi, Mbhashe, Mnquma, and Nkonkobe) between October 2015 and May 2017. This model was based on the Institute for Healthcare Improvement’s (IHI) breakthrough series collaborative model [4]. This was significant, as HIV prevalence in South Africa’s general population was 20.4% at the time of the initiative.

According to the Eastern Cape Department of Health annual report 2017/18, the HIV prevalence among people 15 years and older decreased from 9.9% in 2013/14 to 6.1% in 2017/18 and, similarly in the Amathole district, from 6.6% to 3.1%, respectively. Furthermore, in 2012, the Amathole district had an antenatal HIV seroprevalence of 28.3%, similar to the seroprevalence rate of the Eastern Cape of 28.1% (the 2015 National Antenatal Sentinel HIV and HSV-2 Prevalence Survey in South Africa). The natural environment is similarly diverse, including moist mountainous, well-watered coastal and the semi-arid Karoo, thornveld, succulent, and thicket areas. The district falls within the lowest socio-economic quintile* and is ranked as the fifth most-deprived district in the country, with an estimated medical scheme coverage of 8.7% [5].

The DCST Ministerial Report to the Honourable Minister of Health in 2011 stated that the function of the DCST is to improve the quality of healthcare, as well as health outcomes for mothers, newborns, and children, as a principal component of strengthening the district health system. DCST members are expected to function as a team and individually within their respective disciplines. They are responsible for the following areas of work: quality of clinical services, clinical training, monitoring, evaluation, and improving clinical services. Other responsibilities include supporting district-level organisational activities, health systems and logistics, collaboration, communication and reporting, teaching, and research activities [1,2]. At the district level, the DCST provides technical expertise to strengthen the effectiveness of the health systems and ensure that mothers, newborns, and children are provided with quality healthcare services to reduce maternal and child mortality [1,6].

Furthermore, the functions of the DCST were aligned with the S2S quality improvement programme initiative, which involves organising and implementing activities to improve access to healthcare services and programme monitoring to improve the quality of healthcare service delivery. The complimentary activities include (i) providing clinical training to healthcare professionals (HCPs) to improve their knowledge and ability to deliver the quality of healthcare service, (ii) monitoring and evaluating maternal, child, and women’s health programmes by checking the data submitted by institutions to the District Health Information System (DHIS) and correlating this with the institutional Problem Identification Programme (PIP) data to identify gaps within the datasets, and (iii) ensuring the implementation of the four tiers of clinical governance, supporting the strengthening of management systems, the and provision of supportive supervision to healthcare professionals [6,7]. Therefore, the work of the DCST is essential for delivering a successful health system response and cannot be overemphasised [1,2].

The approved composition of the DCST comprises seven specialists: obstetrician/gynaecologist, advanced midwife, paediatrician, paediatrics nurse, family physician, PHC nurse, and anaesthetist [7].

Since the DCST role requires a good working relationship with the facility, sub-district, district support partners, and the District Health Teams (DHT), it is critical to understand how the district teams are involved in implementing the quality improvement programme. In addition, it is vital to evaluate the DCST’s support that enables the teams to work effectively and efficiently to improve the PMTCT programme in the district.

In addition, there is a need to address the barriers to the implementation of PMTCT services at all levels in South Africa, such as inadequate human resources, poor management systems, a lack of coordination/integration, and a lack of programme management and supportive supervision [8]. Therefore, the views of the HCPs and managers about what can be performed to address these barriers are of great importance.

This study explored the understanding of HCPs and managers on the role of the DCST and how they work together with the district team across management levels, including the supporting partners to improve and strengthen the PMTCT programme performance across the four sub-districts supported by S2S in the Amathole district of the Eastern Cape Province, South Africa.

(*The quintile analysis refers to the percentage of total income received by each twenty percent of households: thus, quintile 1 corresponds to the 20% of households with the lowest income, and quintile 5 to that 20% of highest in-come)

## 2. Methods

We used an interpretive qualitative case study [1,2] to understand the role played by the DCST in the Amathole district in improving the PMTCT programme performance. We used a purposive sampling method which relies on the researcher’s judgement to choose the participant’s potential contribution to this study. We conducted in-depth interviews with eight participants to explore the inter-relationship of the DCST with the district health team and their support of the PMTCT programme.

Eight participants who are members of the quality improvement district teams and provide support across the district were selected. Three Operational Managers (OMs), two Professional Nurses (PNs), two S2S Quality Improvement Advisors, and one member of the District Health Team (DHT) where S2S was providing technical assistance to support the implementation of quality improvement work across four sub-districts (Amahlathi, Mbhashe, Mnquma, and Nkonkobe) in the Amathole district municipality in the Eastern Cape province of South Africa were selected. All the participants were females and in their mid-forties. However, no DCST members were available to participate in the study because of their busy schedules.

Two research assistants were trained on the use of the interview guide that was developed to interview participants. Additional support was provided remotely by video calls with Skype and telephonically. Prior to the commencement of the interviews, the participants were informed of the purpose of the study, agreed to participate of their own free will, and did not reveal their identities to anyone. All the participants were comfortable and agreed to have the interviews conducted in the English language, and the interviews were tape-recorded and transcribed.

The interview guide was developed and pre-tested in three facilities (one community health care and two primary health care) to give an opportunity to seek clarity where necessary and for the participants to provide their suggestions for quality and understanding of the simplicity of the questions. The questionnaires for in-depth interviews were conducted by two trained research assistants who were recruited for this study. Both of them were trained on the interview guide and a pre-test of the interview guide was performed on two previous S2S employees (not counted as part of this study). The aim for this was to review the interview guide and provide necessary suggestions that will give an opportunity to refine the tool before using it for the selected participants. One of the recommendations from the pilot was that one or two Operational Managers (OMs) should be interviewed. This suggestion was very useful because, in facilities where the OMs were interviewed, the quality improvement team members in the facilities were eager to participate in the study because of the buy-in and the directive from their OMs.

The inclusion criteria were members of the quality improvement team who support the implementation of quality improvement work in the facilities supported by the S2S programme in the Amathole district in the Eastern Cape province of South Africa, and those who consent to participate in the study. The exclusion criteria include HCPs who are not involved in the implementation of quality improvement work and who are in non-S2S supported facilities in the Amathole district. Data were analysed thematically by identifying, analysing, and reporting patterns within the data based on the approach developed by Braun and Clarke [9]. Codes were developed based on the emerging themes by reading through the data repeatedly and breaking down the themes, categorising and building them up again in a novel way through elaboration and interpretation.

### Ethical Consideration

The study was approved and ethically cleared by the Senate Research Ethics Committee of the University of the Western Cape, Cape Town, South Africa. Permission was obtained from the Health Department of the Eastern Cape province and the Amathole district management team. The consent forms were signed by all participants and participation was voluntary. The research participants’ identities were protected, and all information was kept confidential. The only involvement of the respondents was their participation in the in-depth interview; therefore, this study was associated with minimal harm.

## 3. Findings

Most interviewees (87.5%) were females in their mid-forties and had been at their respective facilities for at least five years. The mean age of the participants was 45.0 years. The findings of this study were discussed based on three themes: (1) capacity building, which was performed mainly by facilitating the PMTCT in-service training, conducting one-on-one coaching, mentoring and clinical audit as they conduct supportive supervision visits, and based on the identified need, (2) programme performance oversight and monitoring, using facility data to monitor the PMTCT programme performance and to guide the technical support required and, (3) technical support for programme implementation to ensure that the performance improvement plans identified are implemented accordingly.

### 3.1. Capacity Building

One of the key roles of the DCST is to ensure that clinicians have current clinical knowledge, guidelines, protocols, Standard Operating Procedures (SOPs), and have clinical skills that will empower them to provide quality healthcare services to their clients. The DCST provided continuous training to the working staff, which kept them abreast of the latest developments in clinical care and addresses the dynamic changes in the guidelines from time to time. Our data showed that the Amathole district, similar to many other rural districts in South Africa, had high staff turnover, resulting in the loss of valuable knowledge and skills transferred to newly recruited staff who are employed to fill the vacant positions but need to be trained so that they can become successful in their new role. To address this, the DCST trained new staff on new guidelines and policies, which assisted in integrating them into the healthcare system. The participants reported that the DCST played a significant role in clinicians’ one-on-one consultation, which enhanced their clinical skills, knowledge, and work performance to care for and manage the mother and baby pair for the improved PMTCT programme performance in their facilities.

*“The DCST assist in conducting workshops and in-service training on PMTCT related topics. In my clinic, we were trained on proper management of mother and childcare. They support us to implement PMTCT protocol and make sure that we are practising and adhering to the guidelines.”*

(Professional Nurse)

*“I see the role of the DCST in mentoring and coaching us to make sure we implement PMTCT guidelines.”*

(District Health Team Member)

*“In my clinic, we call the DCST anytime when we need support, and they assist us to manage cases that are complicated. They are ready to help us, and they support us”*

(Professional Nurse)

*“My clinic has challenges …. some of them (the staff) are new on the job because many staff leave for better jobs. The DCST assisted by training the staff when we called them.”*

(Professional Nurse)

As reported by the participants, it is imperative that ongoing coaching and mentoring is performed for both the new staff who are expected to perform well in a new job with new responsibilities, as well as to update the knowledge of the existing staff members. Furthermore, it is important to invest in the capacity development of HCPs in order to ensure that NDoH national guidelines, protocols, and SOPs are implemented accordingly, and to meet up with the changes in the PMTCT guidelines so that the PMTCT programme can achieve better clinical outcomes, as recommended by the World Health Organization (WHO) [10].

In addition to the training and coaching of clinicians in the district hospitals, the participants confirmed that the DCST members conduct supportive supervision at the lower-level facilities (community health centres, primary healthcare centres, and gateway clinics). Furthermore, they coached the clinicians on managing routine clinical functions, such as conducting clinical audits. These clinical audits were tailored toward assisting the facilities to identify gaps in the PMTCT performance and to improve the quality of care provided to the mother and child. For example, one of the participants stated

*“I think the DCST supported us to conduct clinical audits and to identify areas of weakness in our clinic. They know the nurses that need help, and they support them to perform their work better.”*

(District Health Team Member)

### 3.2. Programme Performance Oversight and Monitoring

The participants confirmed that the DCST plays a significant role that is complementary to tracking the PMTCT programme performance work performed by the DoH managers and supporting partners, such as the S2S quality improvement advisors. This involves providing programme oversight and monitoring of the PMTCT programme to achieve the desired outcomes. Our findings showed that the DCST support the facilities by using data to identify facilities that are performing poorly, and this informs their decision to schedule facility support visits and plan their supportive supervision visits. This is performed through consultative engagement with the DoH management, facility team, and DoH partners and this is essential for a better understanding of the challenges, inputs, and collecting information that will assist in formulating improvement plans for better outcomes. This works smoothly because they have built strong relationships with all stakeholders, and they are working closely with them. The resultant effect leads to a well-formulated performance improvement plan that is put in place and monitored to address the challenges contributing to poor performance and to achieve better outcomes.

*“They assist us a lot. They help us to see where we are not doing well in terms of our indicators. They also monitor the use of guidelines and see that we manage our patients properly. When we encounter any challenge with our patients, we reach out to them by calling them on phone, and they provide more information and education on how to handle them”*

(Professional Nurse)

*“In my sub-district, the DCST check and cross-check our data when they do their support visits. When there is a reoccurring problem, they assist us and help the staff to understand the issues for not performing well. I know that the DCST organise meetings to analyse data. They also help to review strategies to track patients that are lost to follow-up. For example, tracing of mothers and children who are HIV positive but missed their clinic appointments so that they can be managed according to the DoH protocol”*

(Quality Improvement Advisor)

*“Another thing they assist us with is supporting us to manage cases that are complicated. They advise us whenever we call on them to help us and they support us to plan properly”*

(Operational Manager)

Our data showed that, in addition to ensuring that the guidelines are implemented accordingly, DCSTs are also available to assist clinicians with managing difficult patients. First, this technical assistance empowers HCPs to manage their patients appropriately. Secondly, it also helps build the confidence of HCPs who are now better equipped to handle such patients in the future. For example, the participants alluded to the fact that the DCST ensures that women be tested early in pregnancy and be treated for HIV. They also ensure that women are closely monitored (virally suppressed on treatment), and that the mother–baby pair who missed their clinic appointment are brought back to care and managed accordingly to prevent HIV transmission. One of the participants stated

*“We have seen improvement in early booking, monitoring and recording of viral load in pregnant women that are HIV positive in the clinics. This is as a result of the support we received to use the guidelines and make sure that the mothers were tested early, tested more than once during the antenatal period and ensuring that we start them on ARVs as soon we can. Also, we do not have any record of new infections in newborns because of the support we have received from the DCST. The teams come to our clinic to support us to do our work better …. by assisting us with any challenge we encounter. They also advise us on how we can do our work better.”*

(Professional Nurse)

### 3.3. Technical Support for Programme Implementation

The participants confirmed that the DCST complemented the technical support offered by DoH partners, such as S2S quality improvement advisors, to implement and monitor the change ideas generated during the learning sessions.

*“We (the facility team, DCST and Quality Improvement Advisor) develop a quality improvement plan together. They (DCST) join our monthly data clarification and data cleaning meeting to follow up with the change ideas we have from the S2S quarterly learning sessions, where we also learn about the best practice from other clinics”*.

(Quality Improvement Advisor)

According to the participants, one example of the support provided by the DCST to complement the S2S technical advisors in monitoring the implementation of quality improvement change ideas in the facility is the tracking and tracing of the mother–baby pair (MBP). The DCST used their checklist to ensure that the MBP is traced and returned to care to continue their medication and attend the clinic for a follow-up whenever they miss their appointment. This is very important, especially during breastfeeding, to prevent mother-to-child HIV transmission.

*“Here in my facility, the DCST helps us every time we call.… they help in tracing children who became HIV positive. Even if the child was not delivered here or the child was delivered in a nearby facility, the DCST assist in tracing the child and therefore we are able to initiate the children on ARVs [Antiretroviral therapy] as soon as possible. In my opinion, the DCST assist us to track those that are lost to follow up which is important in our work.”*

(Professional Nurse)

One of the strategies to improve programme performance is to use data for information management. This was another change idea implemented by the facility quality improvement team, where they set aside 30 to 60 min weekly to review their facility’s performance using the programme data. As confirmed by the participants, the DCST leveraged this opportunity to provide technical expertise and delve into the PMTCT performance of the facilities during their site visits.

*“I see that the DCST assist us to analyse different clinic PMTCT data. The DCST provide support and guidance to the managers on different challenges that they experience. They also help us to see where we are not doing well in terms of our indicators.”*

(District Health Team Member)

The participants agreed that, in addition to the technical support provided by the DCST, they also provided additional support for data verification and data analysis purposes to identify gaps in the PMTCT programme; this gave a better understanding of the challenges that might hinder performance. Our data showed that the technical support provided by the DCST team also assisted the facilities in looking at their data in a better way, enabling them to improve their performance. This was evident in the participant’s responses regarding the additional support provided by the DCST.

An overview of the findings from the interviews showed that the overall work of the DCST is to support the district health team and clinicians to do their job better to improve the health systems. In addition, they also assisted the district team in providing quality healthcare services to the community, especially in caring for mothers and children. The participants concluded that the interaction between the DCST, the CQI teams, the district health team, and the DoH management team helped to improve clinical governance and programme performance. This is because of the DCST leadership role, and the technical assistance given to HCWs to support programme implementation aimed at improving the health systems for better-quality care across all disciplines. These are aligned with the core functions of the DCST, which include training, programme monitoring, and clinical governance to enhance and strengthen the district health system, which also complements the continuous quality improvement initiatives implemented by S2S.

From the participant’s responses, the inter-relationship of the district teams and supporting partners, such as the S2S and CQI teams with the DCST, helped provide the necessary support to improve the quality of health services offered within the facilities. They also ensured that clinicians adhered to the implementation of the NDoH guidelines for better clinical outcomes for the clients.

In addition, one of the professional nurses stated that:

*“The DCST guide us to comparing the previous month’s data with the current one to see if we are doing well or not. For example, we will be able to know how many newly positive pregnant women we have in the clinic so that we can compare with those that are already on ARVs and those that are pregnant. When the pregnant mother delivers, we will know the babies that are exposed to HIV and how many new babies or children are infected with HIV”*.

As alluded to by the clinicians, the other key role of the DCST is the technical support provided to the district team to use the facility data in identifying underperforming facilities. Thereafter, remedial actions were developed to improve the facility’s performance and to guide the joint supportive supervision. This support complements the work performed by the S2S quality improvement advisors that support the facilities which are geared towards ensuring that quality improvement plans are monitored to achieve the desired outcome. This finding correlates with the expected role played by the DCST, which is to use their technical expertise to support programme monitoring.

*“We work well with them (DCST), although we do not have all the positions filled in the district. They (DCST) are part of our data meeting and data review meeting every Tuesday and at the end of the month, where we identify gaps in our performance and work together to improve them.”*

(Operational Manager)

Over and above this, the inter-relationship of the team is also seen clearly in the programme performance review meetings held at the facility and district levels. These meetings assist in identifying the best-performing facilities and using the lessons learned to scale up the best practices in underperforming facilities. Additionally, the weekly touch base meeting between the DCST and the district management team serves as an opportunity to disclose challenging areas that need to be addressed to improve programme performance.

As alluded to by the participants, the interrelationship of the DCST with the district health team also ensured the effective implementation of the PMTCT protocols. This begins with prioritising nurses that need to be trained urgently. For example, the Nurse-Initiated Antiretroviral Treatment (NIMART) trained in different units for upcoming training. This is one of the functions of the DCST—to build the capacity of HCPs [11,12] and assist the health facilities in providing quality care to the patients.

## 4. Discussion

More than half of the study participants had been working in the facility for more than five years and were willing to support quality improvement work. This is similar to the findings by Hashish et al., which stated that middle-aged nurses tend to display positive attitudes toward evidence-based practice and quality improvement [13].

The participants’ responses on their understanding of the role of the DCST and their engagement with the district teams at different management levels are geared toward achieving a common goal of improving and strengthening the PMTCT programme performance in the Amathole district of the Eastern Cape Province, South Africa. A good relationship with all stakeholders (DoH and partners) plays a significant role in complementary activities by the DCST, which the participants felt was valuable, especialty for improving healthcare access and quality, as alluded to by the study participants. The participants’ view about the role of the DCST is aligned with the functions stipulated in the ministerial report submitted to the Honourable Minister of Health in 2011 [1], with the overall function of improving the quality of healthcare to achieve a better outcome for mothers, newborns, and children. The themes from the participants were categorised into three groups: building the capacity of HCPs, oversight of programme performance, and monitoring and providing technical support for programme implementation. In addition, their role is cross-cutting and complements the quality improvement work performed by the S2S quality improvement advisors to ensure that the change ideas are implemented and monitored for improved performance.

### 4.1. Supporting Clinical Governance

The DCST has a crucial role in clinical governance by ensuring that the facilities maintain and improve the quality of care, especially for the mother and child. Our study found that the DCST provides leadership and guidance for technical support in implementing the guidelines and protocols. It also provides adequate training to ensure that the HCPs care for their clients and offer quality care to respond to their needs. This finding agrees with Oboirien et al. [14,15], who showed that the role of DCST is to improve clinical governance at the system-wide level. Stonehouse et al. also affirmed that clinical governance ensures that roles are recognised with clear lines of responsibility and accountability for clinical services rendered [16]. This corroborates the participants’ opinion that the DCST plays important role in improving the quality of patient care in the facilities by ensuring the implementation of quality improvement programmes with clear policies for managing risks and procedures to identify and manage poor performance among professionals [16]. This finding shows how the role of the DCST is critical in providing guidance, leadership, and support to the district team to improve programme performance. Likewise, Biyani et al. indicated the seven components of clinical governance: clinical effectiveness, clinical audit, risk management, education and training, patient and public involvement, information, and staff management [17], which are activities that the DCST conducts in the district. These activities clearly show that the DCST roles are broad and interconnected with other stakeholders in the facilities, sub-districts, districts, and provinces. Our study found that there is a need to work with all stakeholders for the successful implementation of clinical governance and for the DCST to be successful in its role. The staff need to be available for training on the guidelines, policies, and procedures to improve their knowledge, competencies, and skills, which will strengthen the health system and lead to better performance at all levels towards achieving Universal Health Coverage (UHC) by 2030 [18].

Although the DCST played a critical role in clinical governance [4], there is not a full complement of these high-skilled specialists in the Amathole district. Therefore, it is vital that the DCST roles are filled, and that the implementation of the programme is monitored closely and regularly evaluated.

### 4.2. Interrelationship of the DCST with the District Health Management Team (DHMT)

Stakeholders’ management and relationships are essential for better coordination of public health activities, and it is essential for collaboration and cooperation to jointly perform better. In addition to improving clinical governance, the DCST also supports programme performance monitoring aimed at improving systems and providing better care across all disciplines, not only the ones catering to mothers, newborns, and children. Our study found that there was a good working relationship between the DoH and the partners, such as S2S. The multidisciplinary team approach helps to look at issues in a more comprehensive way, achieve things quickly, and put a monitoring system in place. Our finding agrees with McKenzie, Harpharm, and Hunter, who conclude that partnership in public health is very important because most improvements recorded in health are not just the consequences of government interventions but a result of collaborated actions from the individuals, communities, organisations, and others that jointly worked together [19,20,21].

Lastly, the DCST also conducted supportive supervision and facilitated the integration and coordination of staff for programme monitoring and improvement, as gathered from the participant’s narratives. Therefore, supportive supervision is essential in improving the health service provision for mothers, newborns, and children, and will strengthen the South African district health system. In addition, the expected role of the DCST stipulates that it needs to implement the four tiers of clinical governance, support the strengthening of management systems, and provide supportive supervision to health professionals [4]. Supportive supervision involves the guiding, monitoring, and coaching of HCPs to ensure the delivery of quality healthcare services and promote teamwork [22], which is a critical part of the DCST work that involves training and guiding the facility teams, with the goal of improving their performance and continuously providing high-quality care to the mother and child and all the clients accessing care in their facility.

### 4.3. Use of Data to Monitor Programme Improvement

The studies by Kagawa Singer et al. [23] and Sibanda et al. [24] stated that good leadership is the key to promoting the use of data and facility performance so that the supervision they provide to the facilities is tailored to address the challenges identified for improved programme performance [24]. The narratives in this study highlighted the need for the facilities to meet each month as a team to review facility data. As a result, the facility performance trends are compared from month to month. As alluded to by the participants, the DCST joins the data review meetings and uses its technical expertise to help the facility team to use data to identify underperformance and jointly develop an improvement plan and monitoring processes.

It cannot be overemphasised how important data is to information management since healthcare workers are overburdened with many demands for data documentation and reporting [25], especially if there are no data-driven frameworks and evaluation tools for integration [24]. This leaves them no time to address healthcare workers’ day-to-day problems that influence their performance. However, the supervisors are better placed to use the findings/report to guide their supportive supervision and provide the required actions to improve facility performance. Therefore, data integration and use are critical in identifying programme gaps to design mitigation plans that will be implemented to improve performance and strengthen the PMTCT cascade.

A study by the U.S. President’s Emergency Plan for AIDS Relief (PEPFAR) concluded that, despite its support for government healthcare systems through the continuous integration of health information systems ensuring that data is readily accessible and data quality is maintained, it is still essential to use data to plan strategies, allocate resources, and procure commodities [25]. This is similar to what we found in our study which showed that the use of data is essential for planning, including human resources for health, training needs, and planning for joint supportive supervision, as demonstrated by the support provided by the DCST.

As found in this study, the technical guidance provided by the DCST and the S2S quality improvement advisors during the facility review meeting involved comparing the previous month’s data with the current one, in order to identify gaps in service delivery and performance Nutley et al. [26] states that one of the best ways to engage data users and data producers to improve the use of health data for health systems is to use people in various job functions and at different levels of the health system to collect, analyse, synthesise, interpret, and use data in decision making. However, if there is no interaction between individuals who design and manage research and information systems, it leads to a breakdown in the programmatic decision-making cycle by the data users [26]. Therefore, if data users and producers work together, they become more aware of the data collection processes and methods, as well as available data sources and data quality. In addition, the data users have the opportunity to address barriers to data use, improve the sharing of data resources, discuss concerns, seek clarification about the data collection process, identify key programmatic questions, link these questions to the data available in their settings, and jointly analyse and interpret data to answer programmatic questions [26]. This approach is used in the quality improvement programme and the DCST to support the facility to use their data to improve their performance as they go for their supervision visits.

In summary, the participants alluded to the fact that the DCST supports clinical governance, provides support to build the capacity of HCPs, provides technical support for programme implementation, and provides oversight for programme performance and monitoring. These (DCST) highly skilled staff support the provision of quality health service delivery by improving HCPs knowledge of new/updated guidelines by providing training and supportive supervision, especially in remote areas where there is no full complement of staff because of high staff turnover due to resignations.

### 4.4. Study Limitations

This study was conducted in one of the 52 district municipalities in South Africa; hence the view of the participants is limited only to the district where the study was conducted. Additionally, because the district did not have a full complement of the DCST and none of the four team members (out of the ideal seven specialists) were available at the time of interviewing the participants due to competing priorities, their views about their roles and responsibilities were not explored. Therefore, we suggest that the DCST could be interviewed for diversity and an in-depth understanding of issues surrounding programme implementation and improvement in the near future.

## 5. Conclusions

The findings of this study show that the role of the DCST is important in improving the quality and service provision of the PMTCT programme and is critical to assist the team at different levels to address challenges encountered and to train and mentor the staff on the PMTCT programme so that they can function at their best. They also work with the team to provide services as stipulated in the national guidelines and use data to inform the technical support and supervision they provide to the facilities. However, it was recommended by the World Health Organization (WHO) and the Institute for Healthcare Improvement (IHI) that a multi-disciplinary team, such as the DCST, is an important component of the health system that facilitates the implementation of quality improvement programmes which will accelerate the PMTCT programme improvement. The strategies implemented by the South African government, including the joint effort by these specialised professionals (DCST) to improve the delivery of quality health services by providing supports, such as the capacity building of HCPs, clinical support on the management of complicated cases, and use of information to manage the PMTCT programmes are key strategies to further improve programme performance, not only for the PMTCT programme but across other programmes, especially in resource-limited settings in Sub-Saharan Africa. In addition, the DCST has a good working relationship with the existing team in the district and at different levels. Their roles and responsibilities cannot be fully achieved without a good working relationship with both the quality improvement team and district health teams because they work better together to ensure that the programme is performing well as systems are put into place to improve the health system toward offering the best care to clients. This includes data used for information management performed by reviewing data as it changes over time to show the performance and what needs to be conducted to perform better. Additionally, Sherr et al. recommended that there is a need for specialist teams, such as the DCST, to engage the facility-level PMTCT staff and managers to understand the system’s performance by using performance data to identify bottlenecks and prioritise areas for improvement [27]. Lastly, we suggest that, in an effort to further reduce the MTCT rate of HIV in the Amathole district in South Africa and sustain the gains made over the past years, more funding should be allocated to recruit and retain the DCST to be able to institutionalise clinical governance at the lowest level of the health system, support HCPs in providing quality health services, and have guiding procedures, protocols, and policies to support its implementation. In the future, it would be beneficial if a study could be conducted to hear from the DCST in other districts and provinces for a better understanding and perspective of attracting these highly skilled specialists and ensuring their roles and responsibilities inform programme performance.
